# Interval Valued Intuitionistic Fuzzy Line Graphs

**DOI:** 10.1186/s13104-022-06124-x

**Published:** 2022-07-15

**Authors:** V. N. Srinivasa Rao Repalle, Keneni Abera Tola, Mamo Abebe Ashebo

**Affiliations:** Department of Mathematics, Wollega University, Nekemte, Ethiopia

**Keywords:** Fuzzy set, Interval-valued intuitionistic fuzzy line graph, Interval-valued intuitionistic fuzzy graph, Isomorphism, Primary 05C72, Secondary 03B20

## Abstract

**Objectives:**

In the field of graph theory, an intuitionistic fuzzy set becomes a useful tool to handle problems related to uncertainty and impreciseness. We introduced the interval-valued intuitionistic fuzzy line graphs (IVIFLG) and explored the results related to IVIFLG.

**Result:**

Some propositions and theorems related to IVIFLG are proposed and proved, which are originated from intuitionistic fuzzy graphs (IVIG). Furthermore, Isomorphism between two IVIFLGs toward their IVIFGs was determined and verified.

## Introduction

After Euler was presented with the impression of Königsberg bridge problem, Graph Theory has become recognized in different academic fields like engineering, social science in medical science, and natural science. A few operations of graphs like line graph, wiener index of graph, cluster and corona operations of graph, total graph, semi-total line and edge join of graphs have been valuable in graph theory and chemical graph theory to consider the properties of boiling point, heat of evaporation, surface tension, vapor pressure, total electron energy of polymers , partition coefficients, ultrasonic sound velocity and internal energy [1–4]. The degree sequence of a graph and algebraic structure of different graphs operations were determined and its result is to the join and corona products of any number of graphs [5]. These operations are not only in classical graphs, they are more useful in fuzzy and generalizations of fuzzy graphs. The real-world problems are often full of uncertainty and impreciseness, Zadeh introduced fuzzy sets and membership degree [6]. Based on Zedeh’s work, Kaufman introduced the notion of fuzzy relations [7]. Then, Rosenfeld [8] followed the Kaufman work and he introduced fuzzy graphs.

Later, Atanassov witnessed that many problems with uncertainty and imprecision were not handled by fuzzy sets(FS) [9]. Then considering this, he added the falsehood degree to membership degree and presented intuitionistic fuzzy sets(IFS) with relations and IFG which is a generalization of FS and their applications [9–11]. In 1993, Mordeson examined the idea of fuzzy line graphs(FLG) for the first time by proofing both sufficient and necessary conditions for FLG to be bijective homomorphism to its FG. And also some theorems and propositions are developed [12].

In 2011 IVFG and its properties were discussed by Akram and Dudek [13]. After that, Akram innovated IVFLG [14]. Afterward, Akram and Davvaz introduced ideas of intuitionistic fuzzy line graphs(IFLG)[15]. Moreover, IFLG and its properties are investigated in [16].

As far as, there exists no research work on the IVIFLG until now. So that, we put forward a new idea and definitions of IVIFLG. The novelty of our works are given as follows: (1) IVIFLG is presented and depicted with an example, (2) many propositions and Theorems on properties of IVIFLG is developed and proved, (3) further, interval-valued intuitionistic weak vertex homomorphism and interval-valued intuitionistic weak line isomorphism are proposed. For the notations not declared in this manuscript, to understand well we recommend the readers to refer [10, 12, 14, 18, 19].

## Main text

We start the section with basic definitions related to IVIFLG. So that, the definitions [1–12] are the well-known definitions used to discuss the main result of this work.

### Definition 1

[17] The graph of the form $G = \left( {V,\;E} \right)$ is an intuitionistic fuzzy graph (IFG) such that

(i) $\sigma _1,\gamma _1:V\rightarrow [0, 1]$ are membership and nonmembership value of vertex set of G respectively and $0\le \sigma _1(v)+ \gamma _1(v)\le 1\ \forall v\in V$,
(ii) $\sigma _2, \gamma _2 : V\times V\rightarrow [0, 1]$ are membership and nonmembership with $\sigma _2(v_iv_j)\le \sigma _1(v_i)\wedge \sigma _1(v_j)$ and $\gamma _2(v_iv_j)\le \gamma _1(v_i)\vee \gamma _1(v_j)$ and $0\le \sigma _2(v_iv_j)+ \gamma _2(v_iv_j)\le 1,\ \forall \ v_iv_j\in E.$

### Definition 2

[20] The line graph L(G) of graph G is defined as

i. Every vertex in *L*(*G*) corresponds to an edge in *G*,
ii. Pair of nodes in *L*(*G*) are adjacent iff their correspondence edges $e_i,e_j\in G$ have a common vertex $v\in G$.

### Definition 3

For $G=(V,E)$ is a graph with $|V|=n$ and $S_i = \{v_i, e_{i_1}, \cdots , e_{i_p}\}$ where $1\le i \le n, 1\le j\le p_i$ and $e_{ij}\in E$ has $v_i$ as a vertex. Then (*S*, *T*) is called intersection graph where $S= \{S_i\}$ is the vertex set of (S, T) and $T= \{S_iS_j | S_i, S_j \in S; S_i \cap S_j\notin \emptyset , i \notin j \}$ is an edge set of (S, T).

*Remark* The given simple graph G and its intersection graph (S, T) are isomorphic to each other($G\cong (S, T)$).

### Definition 4

[14] The line(edge) graph $L(G) = (H,J)$ is where $H= \{\{e\}\cup \{u_e,v_e\}: e\in E, u_e,v_e\in V, e=u_ev_e\}$ and $J= \{S_eS_f : \,\,e,f\in E, e \notin f , S_e \cap S_f\notin \emptyset \}$ with $S_e=\{e\}\cup \{u_e,v_e, e\in E\}.$

### Definition 5

[16] Let $I =(A_1,B_1)$ is an IFG with $A_1 = (\sigma _{A_1}, \gamma_{A_1})$ and $B_1 = (\sigma _{B_1}, \gamma _{B_1})$ be IFS on V and E respectively. Then $(S,T)= (A_2,B_2)$ is an intuitionistic fuzzy intersection graph of *I* whose membership and nonmembership functions are defined as

(i) $$\sigma _{A_2}(S_i) = \sigma _{A_1}(v_i)$$,
  $$\gamma _{A_2}(S_i) = \gamma _{A_1}(v_i)$$, $$\forall \ S_i \in S$$
(ii) $$\sigma _{B_2}(S_i S_j) = \sigma _{B_1}(v_iv_j)$$,
  $$\gamma _{B_2}(S_iS_j) = \gamma _{B_1}(v_iv_j),\,\forall \, S_iS_j \in T.$$

where $A_2 = (\sigma _{A_2}, \gamma _{A_2})$, $B_2 = (\sigma _{B_2}, \gamma _{B_2})$ on S and T respectively.

So, IFG of the intersection graph (*S*, *T*) is isomorphic to I( means, $(S,T)\cong I$ ).

### Definition 6

Consider $L(I^*)=(H,J)$ be line graph of $I^*=(V,E)$. Let $I=(A_1, B_1)$ be IFG of $I^*$ with $A_1 = (\sigma _{A_1}, \gamma _{A_1})$ and $B_1 = (\sigma _{B_1}, \gamma _{B_1})$ be IFS on X and *E* receptively. Then we define the intuitionistic fuzzy line graph $L(I) =(A_2, B_2)$ of I as

(i) $$\sigma _{A_2}(S_e) = \sigma _{B_1}(e)=\sigma _{B_1}(u_ev_e)$$,
  $$\gamma _{A_2}(S_e) = \gamma _{B_1}(e)=\gamma _{B_1}(u_ev_e)$$, for all $$S_e,S_e \in H$$
(ii) $$\sigma _{B_2}(S_eS_f) = \sigma _{B_1}(e)\wedge \sigma _{B_1}(f)$$
  $$\gamma _{B_2}(S_eS_f) = \gamma _{B_1}(e)\vee \gamma _{B_1}(f)$$, $$\forall \, S_eS_f \in J.$$

where $A_2 = (\sigma _{A_2}, \gamma _{A_2})$ and $B_2 = (\sigma _{B_2}, \gamma _{B_2})$ are IFS on H and J respectively.

The $L(I) = (A_2,B_2)$ of IFG I is always IFG.

### Definition 7

[16] Let $I_1=(A_1, B_1)$ and $I_2=(A_2, B_2)$ be two IFGs. The homomorphism of $\psi : I_1 \rightarrow I_2$ is mapping $\psi : V_1 \rightarrow V_2$ such that

(i) $$\sigma _{A_1}(v_i)\le \sigma _{A_2}(\psi (v_i)), \quad \gamma _{A_1}(v_i)\le \gamma _{A_2}(\psi (v_i))$$
(ii) $$\sigma _{B_1}(v_i,v_j)\le \sigma _{B_2} (\psi (v_i)\psi (v_j)),$$
  $$\gamma _{B_1}(v_i,v_j)\le \gamma _{B_2}(\psi (v_i)\psi (v_j)) \ \forall \,v_i\in V_1, v_iv_j\in E_1.$$

### Definition 8

[13] The interval valued FS *A* is characterized by

$$\begin{aligned} A=\{v_i, [\sigma _A^-(v_i),\sigma _A^+(v_i)]:\,v_i\in X\}. \end{aligned}$$

Here, $\sigma _A^-(v_i)$ and $\sigma _A^+(v_i)$ are lower and upper interval of fuzzy subsets *A* of X respectively, such that $\sigma _A^-(v_i)\le \sigma _A^+(v_i) \forall v_i \in V$.

For simplicity, we used IVFS for interval valued fuzzy set.

### Definition 9

Let $A=\{[\sigma _A^-(v),\sigma _A^+(v)]:\,v\in X\}$ be IVFS. Then, the graph $G^*=(V, E$) is called IVFG if the following conditions are satisfied;

$$\begin{aligned}&\sigma _B^-(v_iv_j)\le (\sigma _A^-(v_i)\wedge \sigma _A^-(v_j)\\&\sigma _B^+(v_iv_j)\le \sigma _A^+(v_i)\wedge \sigma _A^+(v_j) \end{aligned}$$

$\forall \,v_i, v_j \in V , \quad \forall \,v_iv_j \in E$ and where $A=[\sigma _A^-,\sigma _A^+]$, $B= [\sigma _B^-,\sigma _B^+]$ is IVFS on V and E respectively.

### Definition 10

Let $G = (A_1, B_1)$ be simple IVFG. Then we define IVF intersection graph $(S,T)=(A_2, B_2)$ as follows:

1. $A_2$ and $B_2$ are IFS of S and T respectively,
2. $$\sigma _{A_2}^-(S_i)=\sigma _{A_1}^-(v_i)$$ and $$\sigma _{A_2}^+(S_i)=\sigma _{A_1}^+(v_i), \forall \, S_i, S_j \in S$$ and
3. $$\sigma _{B_2}^-(S_iS_j)=\sigma _{B_1}^-(v_iv_j)$$,
  $$\sigma _{B_2}^+(S_iS_j)=\sigma _{B_1}^+(v_iv_j), \quad \forall \,S_iS_j \in T$$.

*Remark* The given IVFG G and its intersection graph (S,T) are always isomorphic to each other.

### Definition 11

[14] An interval valued fuzzy line graph(IVFLG) $L(G)=(A_2,B_2)$ of IVFG $G=(A_1,B_1)$ is defined as follows:

- $A_2$ and $B_2$ are IVFS of H and J respectively, where $L(G^*)=(H,J)$
- $$\sigma _{A_2}^-(S_i)=\sigma _{B_1}^-(e) =\sigma _{B_1}^-(u_ev_e)$$,
  $$\sigma _{A_2}^+(S_i)=\sigma _{B_1}^+(e) = \sigma _{B_1}^+(u_ev_e)$$,
- $$\sigma _{B_2}^-(S_eS_f)=\sigma _{B_1}^-(e)\wedge \sigma _{B_1}^-(f)$$,
  $$\sigma _{B_2}^+(S_eS_f)=\sigma _{B_1}^+(e)\wedge \sigma _{B_1}^+(f)$$ for all $$S_e,S_f \in H, S_eS_f\in J.$$

### Definition 12

A graph $I=(A,B)$ with underlying fuzzy set V is IVIFG if

(i) The map $\sigma _A, \gamma _A: V\rightarrow [0,1]$ where $\sigma _A(v_i)=[\sigma _A^-(v_i),\sigma _A^+(v_i) ]$ and $\gamma _A(v_i)=[\gamma _A^-(v_i),\gamma _A^+(v_i) ]$ denote a membership degree and non membership degree of vertex $v_i\in V$, receptively such that $\sigma _A^-(v_i)\le \sigma _A^+(v_i)$, $\gamma _A^-(v_i)\le \gamma _A^+(v_i)$ and $0 \le \sigma _A^+(v_i) +\gamma _A^+(v_i) \le 1\ \forall v_i \in V$,
(ii) The map $\sigma _B, \gamma _B :V\times V \subseteq E \rightarrow [0,1]$ where $\sigma _B(v_iv_j)=[\sigma _B^-(v_iv_j),\sigma _B^+(v_iv_j) ]$ and $\gamma _B(v_iv_j)=[\gamma _B^-(v_iv_j),\gamma _B^+(v_iv_j) ]$ such that
  $$\begin{aligned}&\sigma ^-_{B}(v_iv_j)\le \sigma _A^-(v_i)\wedge \sigma _A^-(v_j)\\&\sigma ^+_{B}(v_iv_j)\le \sigma _A^+(v_i)\wedge \sigma _A^+(v_j)\\&\gamma ^-_{B}(v_iv_j)\le \gamma _A^-(v_i)\vee \gamma _A^-(v_j) \\&\gamma ^+_{B}(v_iv_j)\le \gamma _A^+(v_i)\vee \gamma _A^+(v_j) \end{aligned}$$
  where $$0\le \sigma _{B}^+(v_iv_j)+ \gamma _{B}^+(v_iv_j)\le 1$$ and $$\forall v_iv_j\in E$$.

Now we start the main results of this work by introducing Interval-valued Intuitionistic Fuzzy Line Graph (IVIFLG) and providing examples.

### Definition 13

An interval valued intuitionistic fuzzy line graphs(in short, IVIFLG) $L(I)=(H,J)$ of IVIFG $I=(A_1,B_1)$ is denoted by $L(I)=(A_2,B_2)$ and whose functions of membership and non membership defined as

(i) $A_2$ and $B_2$ are IVIFS of H and J respectively, such that
  $$\begin{aligned} \sigma _{A_2}^-(S_e) \,=\, & {} \sigma _{B_1}^-(e) = \sigma _{B_1}^-(u_ev_e)\\ \sigma _{A_2}^+(S_e)\,=\,& {} \sigma _{B_1}^+(e) \,=\, \sigma _{B_1}^+(u_ev_e)\\ \gamma _{A_2}^-(S_e)= & {} \gamma _{B_1}^-(e) \,=\, \gamma _{B_1}^-(u_ev_e)\\ \gamma _{A_2}^+(S_e)\,=\, & {} \gamma _{B_1}^+(e) \,=\, \gamma _{B_1}^+(u_ev_e) \quad \forall \,S_e\in H. \end{aligned}$$
(ii) The edge set of L(I) is
  $$\begin{aligned}&\sigma _{B_2}^-(S_{e}S_{f})\,=\,\sigma _{B_1}^-(e)\wedge \sigma _{B_1}^-(f)\\&\sigma _{B_2}^+(S_{e}S_{f})\,=\, \sigma _{B_1}^+(e) \wedge \sigma _{B_1}^+(f)\\ \\&\gamma _{B_2}^-(S_{e}S_{f})\,=\, \sigma _{B_1}^-(e) \vee \gamma _{B_1}^-(f) \\&\gamma _{B_2}^+(S_{e}S_{f})\,=\, \gamma _{B_2}^+ (e)\vee \gamma _{B_1}^+(f)\\&\text {for all}\ ,\, S_eS_f \in J. \end{aligned}$$

**Fig. 1:**
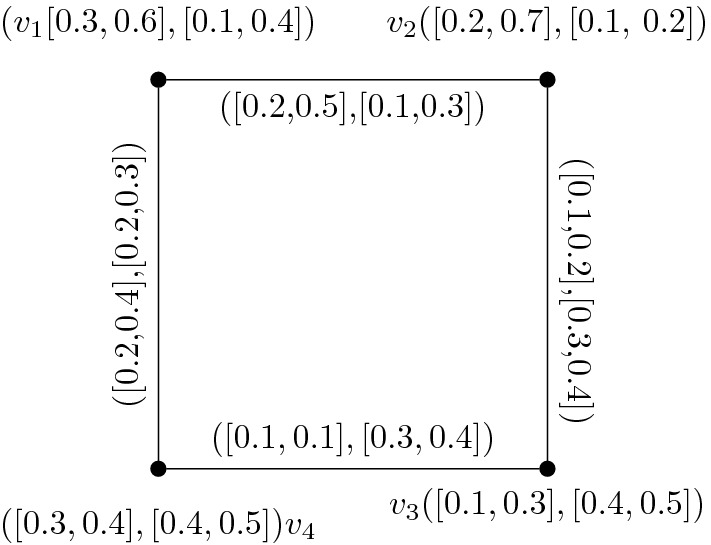
IVIFG I

### Example 14

Given IVIFG $I=(A_1,A_2)$ as shown in Fig. 1.

From the given IVIFG we have

$$\begin{aligned}&\sigma _{A_1} (v_1)= [\sigma _{A_1}^- (v_1),\sigma _{A_1}^+ (v_1)]=[0.3,0.6]\\&\sigma _{A_1}(v_2)= [\sigma _{A_1}^- (v_2),\sigma _{A_1}^+ (v_2)]=[0.2,0.7]\\&\sigma _{A_1} (v_3)= [\sigma _{A_1}^- (v_3),\sigma _{A_1}^+ (v_3)]=[0.1,0.3]\\&\sigma _{A_1}(v_4)= [\sigma _{A_1}^- (v_4),\sigma _{A_1}^+ (v_4)]=[0.3,0.4]\\ \\&\gamma _{A_1} (v_1)= [\gamma _{A_1}^- (v_1),\gamma _{A_1}^+ (v_1)]=[0.1,0.4]\\&\gamma _{A_1} (v_2)= [\gamma _{A_1}^- (v_2),\gamma _{A_1}^+ (v_2)]=[0.1,0.2]\\&\gamma _{A_1} (v_3)= [\gamma _{A_1}^- (v_3),\gamma _{A_1}^+ (v_3)]=[0.4,0.5]\\&\gamma _{A_1}(v_4)= [\gamma _{A_1}^- (v_4),\gamma _{A_1}^+ (v_4)]=[0.4,0.5]\\ \\&\sigma _{B_1}(v_1v_2)= [\sigma _{B_1}^- (v_1v_2),\sigma _{B_1}^+ (v_1v_2)]=[0.2,0.5]\\&\sigma _{B_1}(v_2v_3)= [\sigma _{B_1}^- (v_2v_3),\sigma _{B_1}^+ (v_2v_3)]=[0.1,0.2]\\&\sigma _{B_1}(v_3v_4)= [\sigma _{B_1}^- (v_3v_4),\sigma _{B_1}^+ (v_3v_4)]=[0.1,0.1]\\&\sigma _{B_1}(v_4v_1)= [\sigma _{B_1}^- (v_4v_1),\sigma _{B_1}^+ (v_4v_1)]=[0.2,0.4]\\ \\&\gamma _{B_1}(v_1v_2)= [\gamma _{B_1}^- (v_1v_2),\gamma _{B_1}^+ (v_1v_2)]=[0.1,0.3]\\&\gamma _{B_1}(v_2v_3)= [\gamma _{B_1}^- (v_2v_3),\gamma _{B_1}^+ (v_2v_3)]=[0.3,0.4]\\&\gamma _{B_1}(v_3v_4)= [\gamma _{B_1}^- (v_3v_4),\gamma _{B_1}^+ (v_3v_4)]=[0.3,0.4]\\&\gamma _{B_1}(v_4v_1)= [\gamma _{B_1}^- (v_4v_1),\gamma _{B_1}^+ (v_4v_1)]=[0.2,0.3] \end{aligned}$$

To find IVIFLG $L(I) = (H, J)$ of I such that

$$\begin{aligned}&H=\{v_1v_2=S_{e_1}, v_2v_3=S_{e_2} , v_3v_4=S_{e_3} , v_4v_1=S_{e_4}\} \,\mathrm{and}\\&J=\{S_{e_1} S_{e_2} , S_{e_2}S_{e_3} ,S_{e_23} S_{e_4}, S_{e_4}S_{e_1}\} . \end{aligned}$$

Now, consider $A_2 =[\sigma _{A_2}^-,\sigma _{A_2}^+]$ and $B_2 =[\sigma _{B_2}^-,\sigma _{B_2}^+]$ are IVFS of H and J respectively. Then we have

$$\begin{aligned}&\sigma _{A_2} (S_{e_1})= [\sigma _{B_1}^- (e_1),\sigma _{B_1}^+ (e_1)]=[0.2,0.5]\\&\sigma _{A_2}(S_{e_2})= [\sigma _{B_1}^- (e_2),\sigma _{B_1}^+ (e_2)]=[0.1,0.2]\\&\sigma _{A_2} (S_{e_3})= [\sigma _{B_1}^- (e_3),\sigma _{B_1}^+ (e_3)]=[0.1,0.1]\\&\sigma _{A_2}(S_{e_4})= [\sigma _{B_1}^- (e_4),\sigma _{B_1}^+ (e_4)]=[0.2,0.4]\\ \\&\gamma _{A_2} (S_{e_1})= [\gamma _{B_1}^- (e_1),\gamma _{B_1}^+ (e_1)]=[0.1,0.3]\\&\gamma _{A_2}(S_{e_2})= [\gamma _{B_1}^- (e_2),\gamma _{B_1}^+ (e_2)]=[0.3,0.4]\\&\gamma _{A_2} (S_{e_3})= [\gamma _{B_1}^- (e_3),\gamma _{B_1}^+ (e_3)]=[0.3,0.4]\\&\gamma _{A_2}(S_{e_4})= [\gamma _{B_1}^- (e_4),\gamma _{B_1}^+ (e_4)]=[0.2,0.3]\\ \\&\sigma _{B_2} (S_{e_1} S_{e_2})=[\sigma _{B_1}^-(e_1)\wedge \sigma _{B_1}^-(e_2), \sigma _{B_1}^+(e_1)\wedge \sigma _{B_1}^+(e_2)]=[0.1,0.2]\\&\sigma _{B_2} (S_{e_2} S_{e_3})=[\sigma _{B_1}^-(e_2)\wedge \sigma _{B_1}^-(e_3), \sigma _{B_1}^+(e_2)\wedge \sigma _{B_1}^+(e_3)]=[0.1,0.1]\\&\sigma _{B_2} (S_{e_3} S_{e_4})=[\sigma _{B_1}^-(e_3)\wedge \sigma _{B_1}^-(e_4), \sigma _{B_1}^+(e_3)\wedge \sigma _{B_1}^+(e_4)]=[0.1,0.1]\\&\sigma _{B_2} (S_{e_2} S_{e_3})=[\sigma _{B_1}^-(e_4)\wedge \sigma _{B_1}^-(e_1), \sigma _{B_1}^+(e_4)\wedge \sigma _{B_1}^+(e_1)]=[0.2,0.4]\\ \\&\gamma _{B_2} (S_{e_1} S_{e_2})=[\gamma _{B_1}^-(e_1)\vee \gamma _{B_1}^-(e_2), \gamma _{B_1}^+(e_1)\vee \gamma _{B_1}^+(e_2)]=[0.3,0.4]\\&\gamma _{B_2} (S_{e_2} S_{e_3})=[\gamma _{B_1}^-(e_2)\vee \gamma _{B_1}^-(e_3), \gamma _{B_1}^+(e_2)\vee \gamma _{B_1}^+(e_3)]=[0.3,0.4]\\&\gamma _{B_2} (S_{e_3} S_{e_4})=[\gamma _{B_1}^-(e_3)\vee \gamma _{B_1}^-(e_4), \gamma _{B_1}^+(e_3)\vee \gamma _{B_1}^+(e_4)]=[0.3,0.4]\\&\gamma _{B_2} (S_{e_2} S_{e_3})=[\gamma _{B_1}^-(e_4)\vee \gamma _{B_1}^-(e_1), \gamma _{B_1}^+(e_4)\vee \gamma _{B_1}^+(e_1)]=0.2,0.3] \end{aligned}$$

**Fig. 2:**
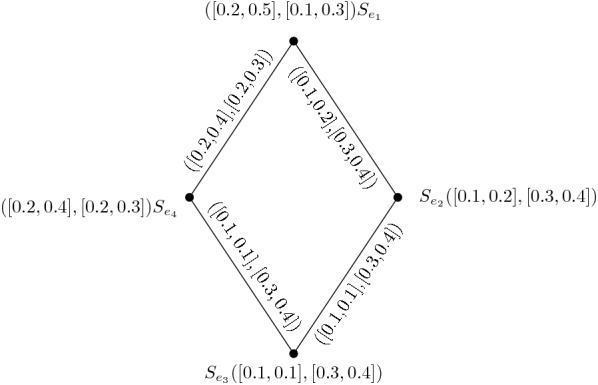
IVIFLG of I

Then L(I) of IVIFG I is shown in Fig. 2.

### Proposition 15

$L(I)=(A_2,B_2)$ is IVIFLG corresponding to IVIFG $I=(A_1,B_1)$.

### Definition 16

A homomorphism mapping $\psi :I_1\rightarrow I_2$ of two IVIFG $I_1 = (M_1, N_1)$ and $I_2 = (M_2, N_2),\ \psi :V_1 \rightarrow V_2$ is defined as

(i) $$\begin{aligned}&\sigma _{ M_1}^-( v_i)\le \sigma _{ M_2}^-( \psi (v_i))\\&\sigma _{ M_1}^+ (v_i)\le \sigma _{ M_2}^+( \psi (v_i))\\&\gamma _{ M_1}^- (v_i)\le \gamma _{ M_2}^-( \psi (v_i))\\&\gamma _{ M_1}^+ (v_i)\le \gamma _{ M_2}^+( \psi (v_i))\\&\text {for all}\ v_i\in V_1. \end{aligned}$$
(ii) $$\begin{aligned}&\sigma _{ N_1}^- (v_iv_j)\le \sigma _{ N_2}^-( \psi (v_i) \psi (v_j)) \\&\sigma _{ N_1}^+ (v_iv_j)\le \sigma _{ N_2}^+( \psi (v_i) \psi (v_j)) \\&\gamma _{ N_1}^- (v_iv_j)\le \gamma _{ N_2}^-( \psi (v_i) \psi (v_j)) \\&\gamma _{ N_1}^+ (v_iv_j)\le \gamma _{ N_2}^+( \psi (v_i) \psi (v_j)) \quad \text {for all}\ v_iv_j\in E_1. \end{aligned}$$

### Definition 17

A bijective homomorphism $\psi :I_1 \rightarrow I_2$ of IVIFG is said to be a weak vertex isomorphism, if

$$\begin{aligned}&\sigma _{M_1} (v_i)=[\sigma _{ M_1}^- (v_i), \sigma _{ M_1}^+ (v_i)]= [\sigma _{ M_2}^- (\psi (v_i)),\sigma _{ M_2}^+ (\psi (v_i))] \\&\gamma _{ N_1} (v_i)=[ \gamma _{ N_1}^- (v_i), \gamma _{ N_1}^+ (v_i)]= [ \gamma _{ N_2}^- (\psi (v_i)), \gamma _{ N_2}^+ (\psi (v_i))] \quad \forall v_i\in V_1. \end{aligned}$$

A bijective homomorphism $\psi :I_1 \rightarrow I_2$ of IVIFG is said to be a weak line isomorphism if

$$\begin{aligned}&\sigma _{B_1} (v_iv_j)=[\sigma _{B_1}^- (v_iv_j), \sigma _{B_1}^+(v_iv_j)]= [\sigma _{B_2}^-( \psi (v_i) \psi (v_j)), \sigma _{B_2}^+( \psi (v_i) \psi (v_j))],\\&\gamma _{B_1} (v_iv_j)=[ \gamma _{B_1}^- (v_iv_j), \gamma _{B_1}^+(v_iv_j)]= [ \gamma _{B_2}^-( \psi (v_i) \psi (v_j)) , \gamma _{B_2}^+( \psi (v_i) \psi (v_j))]\quad \forall v_iv_j\in E_1. \end{aligned}$$

If $\psi : I_1 \rightarrow I_2$ is a bijetctive homomorphism and satisfies Definition 17, $\psi$ is said to be a weak isomorphism of IVIFGs $I_1$ and $I_2$.

### Proposition 18

The IVIFLG L(I) is connected iff IVFG I is connected graph.

### Proof

Suppose *L*(*I*) be connected IVIFLG of I. We need to show that necessary condition. Consider I is disconnected IVIFG. Then there exist at least two nodes of I which are not linked by path, say $v_i$ and $v_j$. If we take one edge *e* in the first component of the edge set of I, then it does not have any edges adjacent to *e* in other components. Then the IVIFLG of I is disconnected and contradicts. Therefore, I is connected.

Conversely, suppose that I is connected IVIFG. Then, there is a path among every pair of nodes. Thus by IVIFLG definition, edges which are adjacent in I are adjacent nodes in IVIFLG. Therefore, each pair of nodes in IVIFLG of I are connected by a path. Hence the proof. $\square$

### Proposition 19

The IVIFLG of IVIFG $K_{1, n }$ is $K_n$ which is complete IVIFG with n nodes.

### Proof

For IVIFG $K_{1, n }$ let us take $v\in V( K_{1, n })$ which is adjacent to every $u_i\in V(K_{1,n})$ where $i=1,2,\cdots , n$. Implies that *v* is adjacent with every $u_i$. Thus, in IVIFLG of $K_{1, n }$, all the vertices are adjacent. This implies that it is complete. Hence the proof. $\square$

### Example 20

Consider the IVIFG $K_{1, 3 }$ whith vertex sets of $V=\{v,v_1,v_2,v_3\}$ and edge sets $E=\{vv_1,vv_2, vv_3\}$ where

$$\begin{aligned}&v=([0.3,0.5],[0.1,0.4]),\, v_1=([0.3,0.4],[0.2,0.5])\\&v_2=([0.5,0.8],[0.1,\,0.2]), \, v_3=([0.1,0.3],[0.5,0.7])\\&e_1=vv_1=([0.2,0.3],[0.3,0.5]),\, e_2=vv_2=([0.2, 0.5],[0.0,0.3])\\&e_3=vv_3=([0.1,0.2],[0.3,0.6]). \end{aligned}$$

Then by definition of IVIFLG, the vertex sets of $L(K_{1, 3 })$ is $V=\{S_{e_1},S_{e_2},S_{e_3}\}$ and $\{S_{e_1}S_{e_2},S_{e_1}S_{e_3}, S_{e_2}S_{e_3}\}$ edge sets where

$$\begin{aligned}&S_{e_1}=([0.2,0.3],[0.3,0.5]),\\&S_{e_2}=([0.2,0.5],[0.0,0.3]),\\&S_{e_3}=([0.1,0.2],[0.2,0.6]),\\&S_{e_1}S_{e_2}=([0.2,0.3],[0.3,0.5]),\\&S_{e_1}S_{e_3}=([0.2,0.3],[0.3,0.5])\\&S_{e_2}S_{e_3}= ([0.1,0.2],[0.2,0.6]). \end{aligned}$$

Here $L (K_{1, 3})$ is complete graph.$K_3.$

**Fig. 3:**
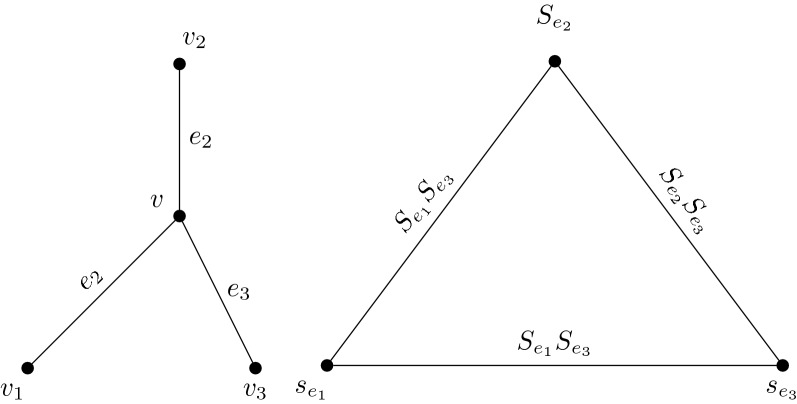
Graphs of $K_{1, 3}$ and $L (K_{1, 3})$

The Fig. 3 depictures the example 20

### Proposition 21

Let *L*(*I*) be IVIFLG of IVIFG of *I*. Then $L(I^*)$ is a line graph of $I^*$ where $I^*=(V,E)$ with underlying set V.

### Proof

Given $I= (A_1, B_1)$ is IVIFG of $I^*$ and $L(I) = (A_2, B_2)$ is IVIFLG of $L(I^*)$.Then

$$\begin{aligned}&\sigma _{A_2}(S_{e})=[\sigma _{A_2}^-(S_{e}), \sigma _{A_2}^+(S_{e})]=[\sigma _{B_1}^-(e), \sigma _{B_1}^+(e) ], \\&\gamma _{A_2}(S_{e})=[\gamma _{A_2}^-(S_{e}),\gamma _{A_2}^+(S_{e})] =[\gamma _{B_1}^-(e), \gamma _{B_1}^+(e) ]\quad \forall \, e\in E. \end{aligned}$$

This implies, $S_e\in H=\{\{e\} \cup \{u_e,v_e\} :\,e\in E, u_e,v_e \in V$ & $e=u_ev_e\}$ if and only if $e\in E.$

$$\begin{aligned}&\sigma _{B_2}(S_{e}S_{f})=[ \sigma _{B_2}^-(S_{e}S_{f}), \sigma _{B_2}^+(S_{e}S_{f})]=[ \sigma _{B_1}^-(e)\wedge \sigma _{B_1}^-(f), \sigma _{B_1}^+(e)\wedge \sigma _{B_1}^+(f)] \\&\gamma _{B_2}(S_{e}S_{f})=[ \gamma _{B_2}^-(S_{e}S_{f}), \gamma _{B_2}^+(S_{e}S_{f})]=[ \gamma _{B_1}^-(e)\vee \gamma _{B_1}^-(f), \gamma _{B_1}^+(e)\vee \gamma _{B_1}^+(f)] \\&\quad \forall \,S_eS_f\in J , \end{aligned}$$

where $J= \{S_eS_f\,{|}\,S_e\cap S_f \notin \emptyset , e,f\in E$ & $e \notin f\}$. Hence, $L(I^*)$ is a line graph of $I^*$. $\square$

### Proposition 22

Let $L(I) = (A_2, B_2)$ be IVIFLG of $L(I^*)$. Then *L*(*I*) is also IVIFLG of some IVIFG $I=(A_1,B_1)$ iff

(i) $$\sigma _{B_2}(S_{e}S_{f})=[\sigma _{B_2}^-(S_{e}S_{f}), \sigma _{B_2}^+(S_{e}S_{f})]= [\sigma _{A_2}^-(S_e) \wedge \,\sigma _{A_2}^-(S_f), \sigma _{A_2}^+(S_e) \wedge \,\sigma _{A_2}^+(S_f) ]$$,
(ii) $$\gamma _{B_2}(S_{e}S_{f})=[\gamma _{B_2}^-(S_{e}S_{f}), \gamma _{B_2}^+(S_{e}S_{f})]= [\gamma _{A_2}^-(S_e) \vee \,\gamma _{A_2}^-(S_f), \gamma _{A_2}^+(S_e) \vee \,\gamma _{A_2}^+(S_f)]\ \forall \, S_e ,S_f \in H,\, S_eS_f \in J$$.

### Proof

Suppose both conditions (*i*) and (*ii*) are satisfied. i.e., $\sigma _{B_2}^-(S_{e}S_{f})= \sigma _{A_2}^-(S_e)\wedge \sigma _{A_2}^-(S_f)$, $\sigma _{B_2}^+(S_{e}S_{f}) =\sigma _{A_2}^+(S_e)\wedge \sigma _{A_2}^+(S_f)$, $\gamma _{B_2}^-(S_{e}S_{f})= \gamma _{A_2}^-(S_e)\vee \gamma _{A_2}^-(S_f)$ and $\gamma _{B_2}^+(S_{e}S_{f}) =\gamma _{A_2}^+(S_e)\vee \gamma _{A_2}^+( S_f)$ for all $S_eS_f\in W$. For every $e\in E$ we define $\sigma _{A_2}^-(S_{e})=\sigma _{A_1}^-(e)$, $\sigma _{A_2}^+(S_{e})=\sigma _{A_1}^+(e)$, $\gamma _{A_2}^-(S_{e})=\gamma _{A_1}^-(e)$ and $\gamma _{A_2}^+(S_{e})=\gamma _{A_1}^+(e)$. Then

$$\begin{aligned} \sigma _{B_2}^-(S_{e}S_{f})&=[\sigma _{B_2}^-(S_{e}S_{f}), \sigma _{B_2}^+(S_{e}S_{f})]\\&=[ \sigma _{A_2}^-(S_e)\wedge \sigma _{A_2}^-(S_f), \sigma _{A_2}^+(S_e)\wedge \sigma _{A_2}^+(S_f) ] \\&=[ \sigma _{B_1}^-(e)\wedge \sigma _{B_1}^-(f), \sigma _{B_1}^+(e)\wedge \sigma _{B_1}^+(f) ].\\ \gamma _{B_2}^-(S_{e}S_{f})&=[\gamma _{B_2}^-(S_{e}S_{f}), \gamma _{B_2}^+(S_{e}S_{f})]\\&=[ \gamma _{A_2}^-(S_e)\vee \gamma _{A_2}^-(S_f), \gamma _{A_2}^+(S_e)\vee \gamma _{A_2}^+(S_f) ] \\&=[ \gamma _{B_1}^-(e)\vee \gamma _{B_1}^-(f), \gamma _{B_1}^+(e)\vee \gamma _{B_1}^+(f) ]. \end{aligned}$$

We know that IVIFS $A_1=([\sigma _{A_1}^-,\sigma _{A_1}^+], [\gamma _{A_1}^-,\gamma _{A_1}^+])$ yields the properties

$$\begin{aligned}&\sigma _{B_1}^-( v_iv_j)\le \sigma _{A_1}^-( v_i)\wedge \sigma _{A_1}^-( v_j) \\&\sigma _{B_1}^+( v_i v_j)\le \sigma _{A_1}^+( v_i)\wedge \sigma _{A_1}^+( v_j)\\&\gamma _{B_1}^-( v_i v_j)\le \gamma _{A_1}^-( v_i)\vee \gamma _{A_1}^-( v_j) \\&\gamma _{B_1}^+( v_i v_j)\le \gamma _{A_1}^+( v_i)\vee \gamma _{A_1}^+( v_j) \end{aligned}$$

will suffice. From definition of IVIFLG the converse of this statement is well known. $\square$

### Proposition 23

An IVIFLG is always a strong IVIFG.

### Proof

It is straightforward from the definition, therefore it is omitted. $\square$

### Proposition 24

Let $I_1$ and $I_2$ IVIFGs of $I_1^*$ and $I_2^*$ respectively. If the mapping $\psi : I_1 \rightarrow I_2$ is a weak isomorphism, then $\psi : I_1^*\rightarrow I_2^*$ is isomorphism map.

### Proof

Suppose $\psi : I_1 \rightarrow I_2$ is a weak isomorphism. Then

$$\begin{aligned}&v\in V_1 \Leftrightarrow \psi (v)\in V_2 \quad \mathrm{and}\\&uv\in E_1 \Leftrightarrow \psi (u)\psi (v)\in E_2. \end{aligned}$$

Hence the proof. $\square$

### Theorem 25

Let $I^*=(V,E)$ is connected graph and consider that $L(I) = (A_2, B_2)$ is IVIFLG corresponding to IVIFG $I = (A_1, B_1)$. Then

1. There is a map $\psi :I\rightarrow L(I)$ which is a weak isomorphism iff $I^*$ a cyclic graph such that
  $$\sigma _{A_1}(v) = [\sigma _{A_1}^-(v) , \sigma _{A_1}^+(v)] =[\sigma _{B_1}^-(e), \sigma _{B_1}^+(e) ],$$
  $$\gamma _{A_1}(v) = [ \gamma _{A_1}^-(v) , \gamma _{A_1}^+(v)]=[ \gamma _{B_1}^-(e), \gamma _{B_1}^+(e) ]$$, $$\forall v \in V , e\in E,$$
  where $$A_1 = ([\sigma _{A_1}^-,\sigma _{A_1}^+], [\gamma _{A_1}^-,\gamma _{A_1}^+])$$ and $$B_1 = ([\sigma _{B_1}^-,\sigma _{B_1}^+], [\gamma _{B_1}^-,\gamma _{B_1}^+])$$.
2. The map $\psi$ is isomorphism if $\psi :I\rightarrow L(I)$ is a weak isomorphism.

### Proof

Consider $\psi :I\rightarrow L(I)$ is a weak isomorphism. Then we have

$$\begin{aligned}&\sigma _{A_1} (v_i)=[\sigma _{ A_1}^- (v_i),\sigma _{ A_1}^+ (v_i)] =[\sigma _{ A_2}^- (\psi (v_i)),\sigma _{ A_2}^+ (\psi (v_i))] \\&\gamma _{ B_1} (v_i)=[ \gamma _{ B_1}^- (v_i), \gamma _{ B_1}^+ (v_i)] = [\gamma _{ B_2}^- (\psi (v_i)), \gamma _{ B_2}^+ (\psi (v_i))] \quad \forall v_i\in V.\\&\sigma _{B_1} (v_iv_j)=[\sigma _{B_1}^- (v_iv_j), \sigma _{B_1}^+(v_iv_j)] = [\sigma _{B_2}^-( \psi (v_i) \psi (v_j)) , \sigma _{B_2}^+( \psi (v_i) \psi (v_j))]\\&\gamma _{B_1} (v_iv_j)=[ \gamma _{B_1}^- (v_iv_j), \gamma _{B_1}^+ (v_iv_j)]= [ \gamma _{B_2}^-( \psi (v_i) \psi (v_j)) , \gamma _{B_2}^+ (\psi (v_i) \psi (v_j))]\quad \forall v_iv_j\in E. \end{aligned}$$

This follows that $I^*= (V, E)$ is a cyclic from proposition 24.

Now let $v_1v_2v_3 \cdots v_nv_1$ be a cycle of $I^*$ where vertices set $V=\{v_1, v_2,\cdots , v_n\}$ and edges set $E=\{v_1v_2, v_2v_3,\cdots , v_nv_1\}$. Then we have IVIFS

$$\begin{aligned}&\sigma _{A_1}(v_i)=[\sigma _{A_1}^-(v_i), \sigma _{A_1}^+(v_i)] =[t_i^-, t_i^+]\\&\gamma _{A_1}(v_i)=[\gamma _{A_1}^-(v_i), \gamma _{A_1}^+(v_i)] =[f_i^-, f_i^+] \end{aligned}$$

and

$$\begin{aligned}&\sigma _{B_1}(v_iv_{i+1})=[\sigma _{B_1}^-(v_iv_{i+1}) \sigma _{B_1}^+(v_iv_{i+1})]=[\iota _i^-, \iota _i^+] \\&\gamma _{B_1}(v_iv_{i+1})=[\gamma _{B_1}^-(v_iv_{i+1}), \gamma _{B_1}^+(v_iv_{i+1})]=[q_i^-, q_i^+], \end{aligned}$$

where $i=1, 2, \cdots , n \,\mathrm{and}\, v_{n+1}=v_1$. Thus, for $t_1^-= t_{n+1}^- , t_1^+= t_{n+1}^+, f_1^-= f_{n+1}^-, f_1^+= f_{n+1}^-$

1 $$\begin{aligned}&\iota _i^-\le t_i^-\,\wedge \,t_{i+1}^-,\nonumber \\&\iota _i^+\le t_i^+\,\wedge \,t_{i+1}^+,\nonumber \\&q_i^-\le f_i^- \,\vee \,f_{i+1}^- \nonumber \\&q_i^+\le f_i^+\,\vee \,f_{i+1}^+. \end{aligned}$$

Now

$$\begin{aligned} & H=\{ S_{e_i} : i= 1, 2, , \cdots , n\} \,\mathrm{and} \quad J=\{ S_{e_i}S_{e_{i+1}} : i= 1, 2, \cdots , n-1\ \}. \end{aligned}$$

And also,

$$\begin{aligned} \sigma _{A_2}(S_{e_i})&=[\sigma _{A_2}^-(S_{e_i}),\sigma _{A_2}^+(S_{e_i}) ]\\&=[\sigma _{B_1}^-(e_i), \sigma _{B_1}^+(e_i)]\\&=[\sigma _{B_1}^-(v_iv_{i+1}), \sigma _{B_1}^+(v_iv_{i+1})]\\&=[\iota ^-_i , \iota ^+_i]\\ \gamma _{A_2}(S_{e_i})&=[\gamma _{A_2}^-(S_{e_i}), \gamma _{A_2}^+(S_{e_i})]\\&=[\gamma _{B_1}^-(e_i), \gamma _{B_1}^+(e_i)]\\&=[\gamma _{B_1}^-(v_iv_{i+1}), \gamma _{B_1}^+(v_iv_{i+1})]\\&=[q^-_i , q^+_i]\\ \sigma _{B_2}^+(S_{e_i}S_{e_{i+1}})&=min \{ \sigma _{B_1}^+(e),\sigma _{B_1}^+(e_{i+1})\}\\&=min\{\sigma _{B_1}^+(v_iv_{i+1}), \sigma _{B_1}^+(v_{i+1}v_{i+2})\}\\&= min\{\iota ^+_i,\iota ^+_{i+1}\}\\ \sigma _{B_2}^-(S_{e_i}S_{e_{i+1}})&=min \{ \sigma _{B_1}^-(e),\sigma _{B_1}^-(e_{i+1})\}\\&=min\{\sigma _{B_1}^-(v_iv_{i+1}), \sigma _{B_1}^-(v_{i+1}v_{i+2})\}\\&= min\{\iota ^-_i,\iota ^-_{i+1}\}\\ \gamma _{B_2}^+(S_{e_i}S_{e_{i+1}})&=max \{ \gamma _{B_1}^+(e),\gamma _{B_1}^+(e_{i+1})\}\\&=max\{\gamma _{B_1}^+(v_iv_{i+1}), \gamma _{B_1}^+(v_{i+1}v_{i+2})\}\\&= max\{q^+_i,q^+_{i+1}\}\\ \gamma _{B_2}^-(S_{e_i}S_{e_{i+1}})&=max \{ \gamma _{B_1}^-(e),\gamma _{B_1}^-(e_{i+1})\}\\&=max\{\gamma _{B_1}^-(v_iv_{i+1}), \gamma _{B_1}^-(v_{i+1}v_{i+2})\}\\&= max\{q^-_i,q^-_{i+1}\} \end{aligned}$$

where $$v_{n+1}=v_1, v_{n+2}=v_2$$, $$\iota ^+_1=\iota ^+_{n+1} , \iota ^-_1=\iota ^-_{n+1}, q^+_{n+1} =\iota ^+_1,$$, $$q^-_{n+1} =q^-_1$$, and $$i=1, 2, \cdots , n$$.

$\psi : V\rightarrow H$ is bijective map since $\psi : I^*\rightarrow L(I^*)$ is isomorphism. And also, $\psi$ preserves adjacency. So that $\psi$ persuades an alternative $\tau$ of $\{1, 2, \cdots , n \}$ which $\psi (v_i)=S_{e_{\tau (i)} }$

and for $e_i=v_iv_{i+1}$ then $\psi (v_i)\psi (v_{i+1})=S_{e_{\tau (i)}} S_{e_{\tau (i+1)}}, i=1,2,\cdots ,n-1$.

Now

$$\begin{aligned}&t_i^-=\sigma _{A_1}^-(v_i)\le \sigma _{A_2}^-(\psi (v_i)) =\sigma _{A_2}^-(S_{e_{\tau (i)} }) =\iota _{\tau (i)}^-,\\&t_i^+=\sigma _{A_1}^+(v_i)\le \sigma _{A_2}^+ (\psi (v_i))= \sigma _{A_2}^+(S_{e_{\tau (i)}}) =\iota _{\tau (i)}^+,\\&f_i^-=\gamma _{A_1}^-(v_i)\le \gamma _{A_2}^-(\psi (v_i)) =\gamma _{A_2}^-(S_{e_{\tau (i)} })=q_{\tau (i)} ^-,\\&f_i^+=\gamma _{A_1}^+(v_i)\le \gamma _{A_2}^+(\psi (v_i)) =\gamma _{A_2}^+(S_{e_{\tau (i)} })=q_{\tau (i)} ^+. \end{aligned}$$

And let $$e_i=v_iv_{i+1}$$,

$$\begin{aligned} \iota _i^-&= \sigma _{B_1}^-(v_iv_{i+1}) \le \sigma _{B_2}^- (\psi (v_i)\psi (v_{i+1}) \\&=\sigma _{B_2}^-(S_{e_{\tau (i)} } S_{e_{\tau (i+1} })) \\&=min\{\sigma _{B_1}^-(e_{\tau (i)}) , \sigma _{B_1}^- (e_{\tau (i+1)} )\} \\&=min\{\iota _{\tau (i)}^-, \iota _{\tau (i+1)}^-\} \\ \iota _i^+&= \sigma _{B_1}^+(v_iv_{i+1}) \le \sigma _{B_2}^+(\psi (v_i)\psi (v_{i+1}) \\&=\sigma _{B_2}^+(S_{e_{\tau (i)} } S_{e_{\tau (i+1} })) \\&=min\{\sigma _{B_1}^+(e_{\tau (i)}) , \sigma _{B_1}^+ (e_{\tau (i+1)} )\} \\&=min\{\iota _{\tau (i)}^+, \iota _{\tau (i+1)}^+\} \\ q_i^-&= \gamma _{B_1}^-(v_iv_{i+1}) \le \gamma _{B_2}^- (\psi (v_i)\psi (v_{i+1}) \\&=\gamma _{B_2}^-(S_{e_{\tau (i)} } S_{e_{\tau (i+1} })) \\&=max\{\gamma _{B_1}^-(e_{\tau (i)}) , \gamma _{B_1}^-( e_{\tau (i+1)} )\} \\&=max\{q_{\tau (i)}^-, q_{\tau (i+1)}^-\} \\ q_i^+&= \gamma _{B_1}^+(v_iv_{i+1}) \le \gamma _{B_2}^+(\psi (v_i)\psi (v_{i+1}) \\&=\gamma _{B_2}^+(S_{e_{\tau (i)} } S_{e_{\tau (i+1} })) \\&=max\{\gamma _{B_1}^+(e_{\tau (i)}) , \gamma _{B_1}^+( e_{\tau (i+1)} )\} \\&=max\{q_{\tau (i)}^+, q_{\tau (i+1)}^+\} \quad for \,i=1,2,\cdots , n . \end{aligned}$$

Which implies,

2 $$\begin{aligned}&t^-_i\le \iota ^-_{\tau (i)} , \quad t_i^+\le \iota _{\tau (i)}^+ \nonumber \\&f^-_i\le q^-_{\tau (i)}, \quad f_i^+\le q^+_{\tau (i)} \end{aligned}$$

and

3 $$\begin{aligned}&\iota ^-_i\le min\{ \iota ^-_{\tau (i)}, \iota ^-_{\tau (i+1)}\} , \quad \iota ^+_i\le min\{ \iota ^+_{\tau (i)}, \iota ^+_{\tau (i+1)}\} \nonumber \\&q^-_i\le max\{ q^-_{\tau (i)}, q^-_{\tau (i+1)}\} , \quad q^+_i\le max\{ q^+_{\tau (i)}, q^+_{\tau (i+1)}\}. \end{aligned}$$

Thus from the above equations, we obtain $\iota _{i}^-\le \iota _{\tau (i)}^-, \iota ^+_i\le \iota ^+_{\tau (i)}, q^-_i\le q^-_{\tau (i)}$ and $q^+_i\le q^+_{\tau (i)}$. and also $\iota ^-_{\tau (i)} \le \iota ^-_{\tau (\tau (i))}, \iota ^+_{\tau (i)} \le \iota ^+_{\tau (\tau (i))}, q^-_{\tau (i)} \le q^-_{\tau (\tau (i))}$and $q^+_{\tau (i)} \le q^+_{\tau (\tau (i))}$. By proceeding this process, we get

$$\begin{gathered} \iota _{i}^{ - } \le \iota _{{\tau (i)}}^{ - } \le \cdots \le \iota _{{\tau ^{k} (i)}}^{ - } \le \iota _{i}^{ - } \hfill \\ \iota _{i}^{ + } \le \iota _{{\tau (i)}}^{ + } \le \cdots \le \iota _{{\tau ^{k} (i)}}^{ + } \le \iota _{i}^{ + } \hfill \\ q_{i}^{ - } \le q_{{\tau (i)}}^{ - } \le \cdots \le q_{{\tau ^{k} (i)}}^{ - } \le q_{i}^{ - } \hfill \\ q_{i}^{ + } \le q_{{\tau (i)}}^{ + } \le \cdots \le q_{{\tau ^{k} (i)}}^{ + } \le q_{i}^{ + } \hfill \\ \end{gathered}$$

where $\tau ^{k+1}$ is the identity function. It follows $\iota ^-_{\tau (i)} = \iota ^-_{\tau (\tau (i))}, \iota ^+_{\tau (i)} =\iota ^+_{\tau (\tau (i))}, q^-_{\tau (i)} = q^-_{\tau (\tau (i))}$ and $q^+_{\tau (i)} = q^+_{\tau (\tau (i))}$. Again, from Eq. 3, we get

$$\begin{aligned}&\iota ^-_{i}\le \iota ^-_{\tau (i+1)}=\iota ^-_{i+1}, \quad \iota ^+_{i}\le \iota ^+_{\tau (i+1)} =\iota ^+_{i+1}\\&q^-_{i}\le q^-_{\tau (i+1)} =q^-_{i+1}, q^+_{i} \le q^+_{\tau (i+1)} =q^-_{i+1} . \end{aligned}$$

This implies for all $i=1,2,\cdots ,n$, $\iota ^-_{i}=\iota ^-_{1} , \iota ^+_{i}=\iota ^+_{1}, q^-_{i}=q^-_{1}$ and $q^+_{i}=q+_{1}$. Thus, from Eqs. 1 and 2 we obtain

$$\begin{gathered} \iota _{1}^{ - } = \cdots = \iota _{n}^{ - } = t_{1}^{ - } = \cdots = t_{n}^{ - } \hfill \\ \iota _{1}^{ + } = \cdots = \iota _{n}^{ + } = t_{1}^{ + } = \cdots = t_{n}^{ + } \hfill \\ q_{1}^{ - } = \cdots = q_{n}^{ - } = f_{1}^{ - } = \cdots = f_{n}^{ - } \hfill \\ q_{1}^{ + } = \cdots = q_{n}^{ + } = f_{1}^{ + } = \cdots = f_{n}^{ + } {\mkern 1mu} . \hfill \\ \end{gathered}$$

Hence the proof. $\square$

### Theorem 26

The IVIFLG of connected simple IVIFG *I* is a path graph iff *I* is path graph.

### Proof

Consider a path IVIFG I of with $|V(I)|=k$. This implies I is $P_k$ path graph and $|E(I)|=k-1$. Since the edge sets of I is the vertices set of IVIFLG *L*(*I*) , clearly *L*(*I*) is a path graph with number of vertices $|V(L(I))|=k-1$ and $|E(L(I))|=k-2$ edges. Then it’s a path graph.

Conversely, suppose *L*(*I*) is a path graph. This implies that each degree of vertex $v_i\in I$ is not greater than two. If one of the degrees of vertex $v_i\ in I$ is greater than two, then the edges *e* which incident to $v_i \in I$ would form a complete subgraph of IVIFLG *L*(*I*) of more than two vertices. Therefore, the IVIFG *I* must be either cyclic or path graph. But, it can’t be the cyclic graph since a line graph of the cyclic graph is the cyclic graph. Hence the proof. $\square$

## Limitations

- This paper introduces only the new concept of IVIFLG which is the extension of IFLG.
- We focused only on some properties of IVFLG and not all properties are mentioned.
- Due to uncertainty and imprecise many real-world problems like networks communication, machine learning, data organization, traffic light control, computational devices, medical diagnosis, decision making, and the flow of computation is difficult to solve without using IFS, IVIF models it has become rapidly useful in the world. But, in this paper the application part is not included.

## Data Availability

Not applicable.
